# LRD-Inst: a lightweight and robust dual-branch framework for instance segmentation of mixed bagged and unbagged apples

**DOI:** 10.3389/fpls.2026.1871083

**Published:** 2026-07-15

**Authors:** Chunyan Zhu, Xilei Zeng, Wenquan Liu, Qun Song

**Affiliations:** 1 School of Intelligent Science and Engineering, Xi’an Peihua University, Xi’an, China; 2 School of Automation, Northwestern Polytechnical University, Xi’an, China

**Keywords:** dual-branch architecture, fruit-picking robot, instance segmentation, lightweight network, state space model

## Abstract

**Introduction:**

Amid global labor shortages, automated harvesting robots are essential for enhancing agricultural productivity, with robust instance segmentation serving as the core vision task. However, existing methods fail to balance high-fidelity boundary delineation and real-time efficiency under severe visual degradations caused by protective fruit bagging and dense canopy occlusions.

**Methods:**

To resolve these limitations, LRD-Inst, a lightweight and robust dual-branch instance segmentation framework, is introduced for unstructured orchards and resource-constrained edge platforms. The architecture explicitly decouples feature extraction: a spatial pathway utilizes Parallel Hierarchical Enhancement Blocks (PHEB) and Frequency-Decoupled Spatial Pyramids (FDSP) to safeguard high-frequency boundary cues, while a contextual branch embeds a High-frequency Detour State Space Model (HDSSM) to capture long-range global dependencies for obscured targets. A Spatially-Refined Adaptive Fusion (SRAF) module bridges these pathways, optimized via an Area-Stratified Dice (AS-Dice) loss to reinforce small-target geometric fidelity.

**Results:**

Extensive experiments on a mixed-apple dataset demonstrate that LRD-Inst achieves a primary Average Precision (AP) of 0.568 with only 3.43 M parameters and 9.12 GFLOPs, outperforming contemporary baselines including the YOLOv8-YOLOv26 families and RTMDet. The model operates at 45.4 FPS on an NVIDIA RTX 3060 GPU.

**Discussion:**

LRD-Inst establishes an optimal equilibrium between accuracy and efficiency, providing a highly deployable solution for autonomous agricultural robotics. The source code is available at https://github.com/ly27253/LRD-Inst.

## 1 Introduction

Apples represent one of the most economically vital fruit crops worldwide. In modern agriculture, transitioning from labor-intensive manual harvesting to intelligent robotic systems is paramount for scaling orchard production (Zeng et al., 2025a, 2025b). Over the past decades, computer vision methodologies for automated apple recognition have transitioned significantly from traditional hand-crafted feature extraction architectures—which rely heavily on heuristic color index segmentation, texture gradients, and geometric thresholding—to deep learning-based object detection frameworks. Although standard bounding-box detectors facilitate rapid target localization, they fundamentally fail to deliver the geometric fidelity required by modern high-density dwarf-canopy cultivation systems. In these densely cluster-structured orchard environments, automated harvesting demands more than coarse object presence detection; it requires exact spatial comprehension of fruit boundaries and individual configurations.

Within autonomous harvesting frameworks, vision-based instance segmentation plays an indispensable role. By delivering pixel-level localization and precise morphological contours, it serves as the foundational visual prerequisite for fruit recognition, dynamic obstacle avoidance, and robotic grasping in unstructured environments (Liu and Liu, 2024; Rajendran et al., 2024; Sapkota and Karkee, 2024). This fine-grained spatial perception represents an absolute necessity for robotic end-effectors to accurately estimate 3D poses, calculate precise grasp centerlines, and execute collision-free picking trajectories. Without pixel-level mask boundaries, robotic manipulators cannot effectively differentiate heavily overlapping fruit clusters or avoid structural branches, inevitably leading to fruit bruising or mechanical damage during detachment. Therefore, conducting research into high-fidelity, real-time instance-level recognition is imperative to bridge the critical operational chasm between abstract visual perception and non-destructive physical manipulation.

However, executing such sophisticated pixel-level processing directly in agricultural fields requires embedding these deep learning-based perception frameworks onto the resource-constrained edge devices inherent to autonomous mobile robots. This operational reality demands high segmentation accuracy under strict computational budgets, prompting a widespread academic shift toward structural optimization. Early lightweight models primarily reduced network redundancy via channel pruning or depthwise separable convolutions (Mahmud et al., 2024; Zawish et al., 2024; Qiu et al., 2021), which frequently suffered from degraded feature expressiveness. More recently, researchers favor one-stage architectures equipped with streamlined backbones, neural architecture search (Li et al., 2021), lightweight attention (Guan et al., 2023; Shi et al., 2024), and structural re-parameterization (Tang et al., 2025) over computationally heavy two-stage models like Mask R-CNN (He et al., 2017). Specifically, architectures such as RTMDet (Lyu et al., 2022) and the YOLO family (YOLOv8–YOLOv26) have dominated real-time instance segmentation through anchor-free prediction, simplified pyramidal connections, and decoupled heads (Jocher et al., 2023; Wang et al., 2024a; Tian et al., 2025; Lei et al., 2025). While these customized networks provide a robust trade-off between segmentation accuracy and inference speed, achieving high-fidelity boundary delineation under extreme computational constraints remains a persistent challenge in complex orchard scenarios.

Despite significant progress, real-world orchard environments present distinct, underexplored challenges in existing datasets and models (Pacheco-Ruiz et al., 2025). A prominent challenge arises from the modern cultivation practice of fruit bagging. To protect against pests and enhance fruit finish, apples are frequently enclosed in protective plastic or paper bags during their growth cycle. This practice introduces extreme visual degradation: plastic bags generate severe specular reflections, obscure natural textures, and significantly mute color distributions (Sun et al., 2019). When mature bagged apples are intermixed with unbagged fruits under varying canopy illumination and dense foliage occlusion, target intra-class variance increases drastically (Hou et al., 2023). Constrained by limited receptive fields and shallow semantic extraction, conventional lightweight models fail to maintain semantic coherence when textures are heavily distorted by plastic films, frequently yielding fragmented masks, blurred boundaries, or missed detections (You et al., 2026; Hu et al., 2026). Effectively segmenting highly variable targets requires balancing fine-grained spatial preservation with long-range semantic contextualization. Feature decoupling and spatial–semantic separation are widely studied strategies for improving structure awareness (Yin et al., 2021). Because capturing local detail is critical for sharp boundary prediction, dilated convolutions are extensively adopted in lightweight modules to expand receptive fields without introducing additional parameters. While cascading dilated convolutions or employing hierarchical attention (Chen et al., 2022) are proven strategies, parallel multi-scale architectures offer a compelling alternative paradigm. These parallel structures simultaneously extract heterogeneous features across multiple receptive fields, actively preserving fine-grained spatial cues that might otherwise attenuate during deep sequential transmission. Furthermore, guided aggregation modules are frequently utilized to selectively propagate complementary cues across layers, yielding finer boundaries and enhanced global consistency (Huang et al., 2025).

On the other hand, robust structural recognition of severely occluded or bagged targets demands potent global semantic modeling (Wang et al., 2025). Although transformer-based self-attention mechanisms provide global context, their quadratic computational complexity hinders edge deployment (Liu et al., 2025). Recently, State Space Models (SSMs), particularly Mamba (Gu and Dao, 2023), have emerged as a revolutionary sequence modeling paradigm, demonstrating an exceptional capability to capture long-range dependencies with linear complexity. However, synergistically integrating the global reasoning of SSMs with the high-resolution detail preservation of CNNs into a unified, lightweight dual-branch architecture (Zhang et al., 2025) for dense agricultural prediction remains an open problem.

Furthermore, scale imbalance is a persistent bottleneck in dense orchard scenes (Cui et al., 2025). Small, distant fruits contribute weak gradients, while large, foreground objects dominate the optimization process. Prior efforts have employed multi-scale pyramids (Lin et al., 2017; Ghiasi et al., 2019), deformable receptive fields (Dai et al., 2017), or adaptive weighting losses (Luo et al., 2023; Liu et al., 2024a) to mitigate this issue. However, these strategies often treat scales independently or lack unified gradient coordination, making it difficult to maintain small-object fidelity without sacrificing global consistency.

To overcome these critical bottlenecks, a novel lightweight and robust dual-branch instance segmentation network, LRD-Inst, is proposed for highly unstructured orchard environments. Recognizing that computational efficiency and precise segmentation are prerequisites for agricultural deployment, the framework adopts a dual-pathway topology that explicitly decouples spatial detail preservation from semantic context extraction. For spatial and multi-scale feature representation, the Parallel Hierarchical Enhancement Block (PHEB) and Frequency-Decoupled Spatial Pyramid (FDSP) are designed to utilize parallel dilated convolutions. Although the hierarchical dilated layout in FDSP draws architectural inspiration from the efficient structures of ESPNet (Mehta et al., 2018), its feature decomposition routing is specifically re-engineered based on fruit segmentation demands to actively isolate and retain high-frequency boundary cues while enlarging the receptive field. Concurrently, to address the severe texture degradation caused by fruit bagging, the High-frequency Detour State Space Model (HDSSM) is introduced into the contextual branch. While the underlying state space formulation adapts the linear-complexity sequence modeling of Mamba (Gu and Dao, 2023) to capture holistic scene-level context efficiently, generic Mamba implementations tend to suppress high-frequency spatial details vital for small-target localization; thus, HDSSM uniquely incorporates a custom high-frequency detour mechanism to counteract this semantic attenuation and preserve micro-scale details. To seamlessly bridge these pathways, a Spatially-Refined Adaptive Fusion (SRAF) module establishes an efficient bidirectional interaction mechanism. Distinct from generic gated fusion networks that inspire its weighting strategy, SRAF is explicitly engineered to bridge the architectural chasm between long-range contextual dependencies and local spatial details, thereby reinforcing detail preservation with minimal computational overhead.

**Figure 1:**
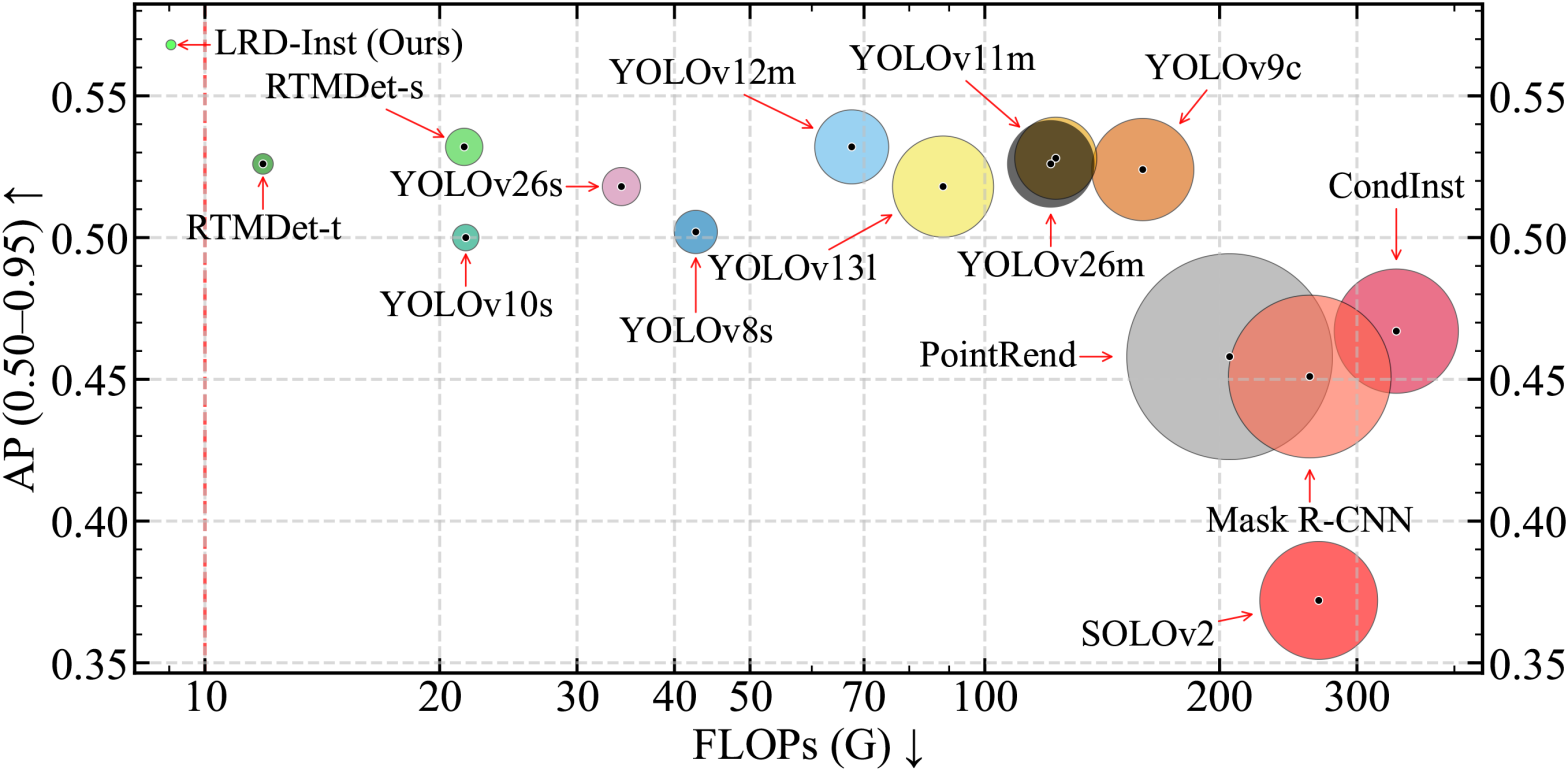
Trade-off analysis of RED-Inst against state-of-the-art models on instance segmentation accuracy, Params, and FLOPs (larger bubbles indicate more Params).

The primary contributions of this study are summarized as follows:

1. SSM-Based Dual-Branch Framework: The proposed LRD-Inst leverages a parallel dual-pathway topology integrating lightweight components (PHEB, parallel FDSP, and SRAF) with a linear-complexity HDSSM module. This paradigm captures global context to infer obscured targets while preserving high-frequency multi-scale details and mitigating information loss inherent in deep serial architectures.
2. Area-Stratified Optimization: An AS-Dice loss featuring area-sensitive reweighting is formulated to balance optimization across scales, explicitly amplifying gradients for diminutive targets to enhance small-object fidelity.
3. Dataset Construction and SOTA Evaluation: A balanced real-world mixed-apple dataset capturing specular interference and high intra-class variance is established. Extensive benchmarks demonstrate that LRD-Inst delivers state-of-the-art instance segmentation performance with an optimal accuracy efficiency trade-off visualized in **Figure 1**.

The rest of this paper is organized as follows: Section 2 introduces the proposed LRD-Inst architecture and its key components; Section 3 describes the apple instance segmentation dataset; Section 4 presents the experimental setup and results; Section 5 discusses the findings; and Section 6 concludes the paper.

## 2 Methodology

### 2.1 Overview

**Figure 2:**
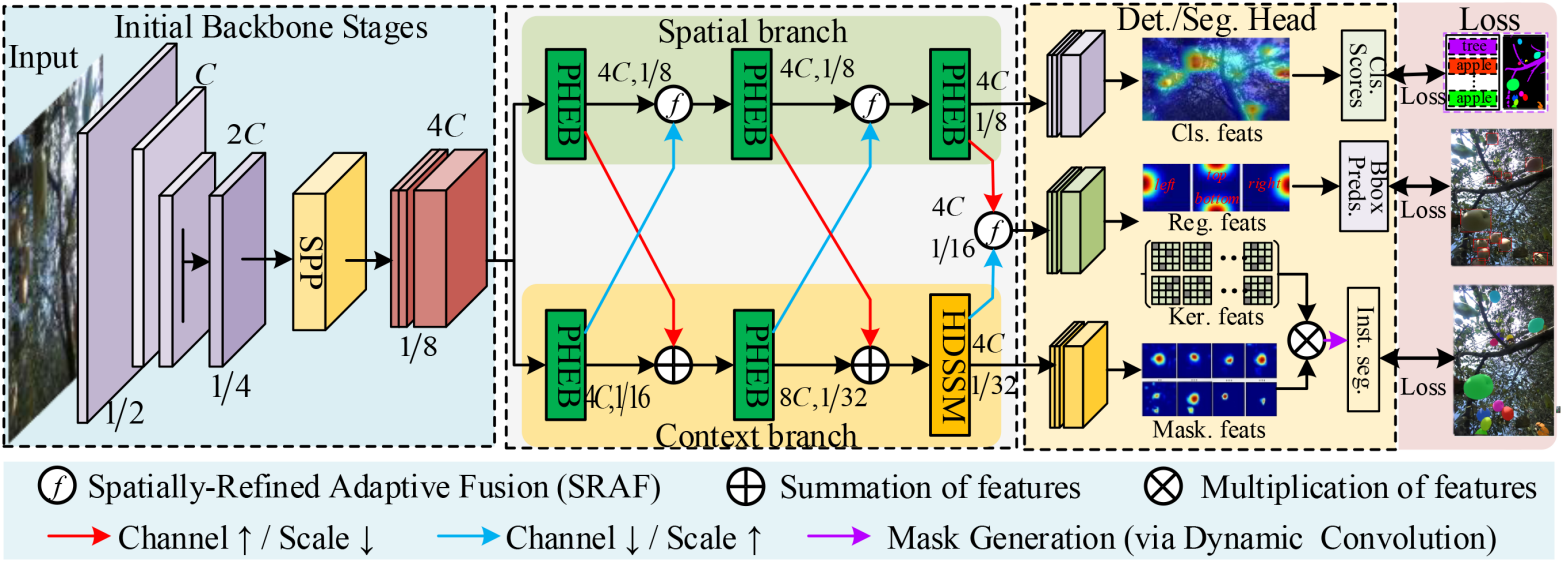
Overall architecture of the LRD-Inst framework. The diagram illustrates the dual-branch topology, the integration of PHEB and FDSP for multi-scale extraction, the HDSSM for global semantic modeling, and the SRAF module for cross-branch fusion.

The LRD-Inst dual-branch instance segmentation framework is introduced to address the visual complexities of unstructured orchards (**Figure 2**). Following backbone extraction via an initial front-end processing stage—which sequentially employs three convolutional layers for shallow feature aggregation and a cross-stage partial downsampling block integrated with channel attention to reduce spatial resolution while preserving descriptive capacity—and Spatial Pyramid Pooling (SPP), the network bifurcates into high-resolution spatial and deep contextual pathways. Both branches utilize Parallel Hierarchical Enhancement Blocks (PHEB, Sec. 2.3)—driven by Frequency-Decoupled Spatial Pyramids (FDSP, Sec. 2.2)—for detail-preserving multi-scale extraction. The contextual branch incorporates a High-frequency Detour State Space Model (HDSSM, Sec. 2.4) to efficiently capture long-range global dependencies. To bridge the semantic gap, a bidirectional interaction mechanism enables spatial cues to enhance the contextual pathway, while deep semantics adaptively refine spatial features via Spatially-Refined Adaptive Fusion (SRAF, Sec. 2.5). Finally, a unified head (Sec. 2.6) generates instance-specific masks through dynamic convolution. The framework is optimized via a composite objective featuring an Area-Stratified Dice (AS-Dice) loss, ensuring scale-invariant boundary delineation for real-time deployment.

### 2.2 Frequency-decoupled spatial pyramid

**Figure 3:**
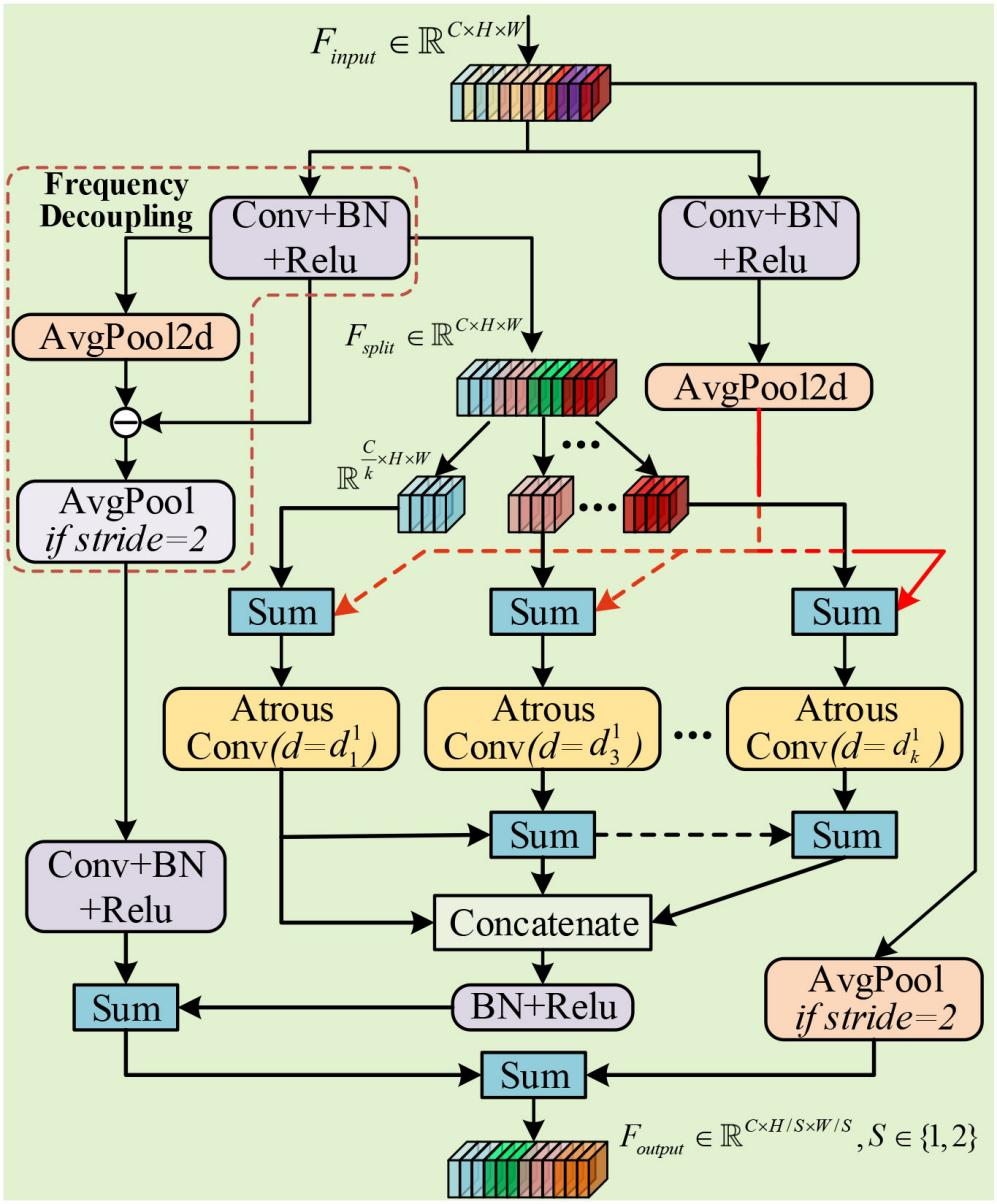
Architecture of the Frequency-Decoupled Spatial Pyramid (FDSP) module. High-frequency spatial details are explicitly isolated via a parallel detour, while mainstream features undergo hierarchical multi-scale extraction to aggregate deep semantic context.

In practical field environments, protective fruit bagging, specular reflections on fruit surfaces, and complex weed backgrounds introduce massive high-frequency noise that severely degrades edge distinctiveness. Traditional spatial pyramids using standard dilated convolutions inherently lack grain-preservation pathways, causing fragile boundary cues of adjacent or bagged apples to blend into the canopy backdrop. The Frequency-Decoupled Spatial Pyramid (FDSP) module is designed to aggregate multi-scale semantic context while explicitly preserving fine-grained edge and texture details (**Figure 3**). Diverging from traditional spatial pyramids, FDSP employs a decoupling architecture: mainstream features undergo hierarchical multi-scale extraction, while high-frequency spatial cues are isolated and adaptively injected via a parallel detour.

Given an input feature map $F_{\text{input}}\in {\mathbb{R}}^{C\times H\times W}$, two parallel 1×1 convolutional projections initially generate a primary feature $F_{\text{split}}$ and an auxiliary residual $F_{a}$, as defined in Equation 1:

(1) $$F_{\text{split}}=f_{1\times 1}(F_{\text{input}}),\;F_{a}=f_{1\times 1}^{\text{add}}(F_{\text{input}})$$

To separate local details from structural semantics, $F_{\text{split}}$ is processed through a 3×3 average pooling operation (AvgPool2d) serving as a low-pass filter to yield *F_p_*, while the auxiliary residual *F_a_* is similarly pooled to generate its low-frequency counterpart $F_{a}^{\text{low}}$. The high-frequency detail residual $F_{\text{high}}$ is then Extracted via element-wise subtraction, formulated as Equation 2:

(2) $$F_{p}=\text{AvgPool}2\text{d}(F_{\text{split}}),\;F_{\text{high}}=F_{\text{split}}-F_{p},\;F_{a}^{\text{low}}=\text{AvgPool}2\text{d}(F_{a})$$

Concurrently, $F_{\text{split}}$ is partitioned into k subgroups along the channel dimension. To extract multi-scale context while mitigating gridding artifacts, each subgroup integrates the low-frequency auxiliary residual $F_{a}^{\text{low}}$ before passing through an Atrous Convolution $D_{3\times 3}(\cdot ;d_{i})$ with a dilation rate $d_{i}$, expressed in Equation 3:

(3) $$Y^{i}=D_{3\times 3}(F_{\text{split}}^{i}+F_{a}^{\text{low}};d_{i}),\;i=1,2,\cdots ,k$$

Cross-receptive-field interaction is facilitated through sequential hierarchical feature fusion, where branch outputs are progressively aggregated, as given by Equation 4:

(4) $${\tilde{Y}}^{i}=Y^{i}+{\tilde{Y}}^{i-1},\;{\tilde{Y}}^{1}=Y^{1}$$

The resulting representations are concatenated and projected to form the expanded contextual feature $F_{\text{exp}}=f_{1\times 1}^{\text{exp}}(\delta (\text{BN}(\text{Concat}({\tilde{Y}}^{1},\cdots ,{\tilde{Y}}^{k}))))$.

Crucially, high-frequency details (*F*_high_) bypass the dilated bottlenecks to prevent information degradation. During downsampling (*stride* = 2), *F*_high_ undergoes adaptive spatial alignment and channel projection to form ${\bar{F}}_{thigh}$, which is then fused with the mainstream representation, described in Equation 5:

(5) $$F_{\text{fused}}=F_{\text{exp}}+{\bar{F}}_{\text{high}}$$

Finally, a global residual connection from $F_{\text{input}}$ is added to $F_{\text{fused}}$, incorporating adaptive spatial pooling for resolution reduction where necessary. This yields the ultimate output $F_{\text{output}}\in {\mathbb{R}}^{C\times H/S\times W/S}$, with S∈{1,2} denoting the stride, yielding the ultimate output on Equation 6:

(6) $$F_{\text{output}}=F_{\text{fused}}+{\text{AvgPool}}_{S}(F_{\text{input}})$$

In summary, FDSP achieves efficient semantic abstraction while actively safeguarding high-frequency textures, ensuring both global structural coherence and crisp boundary delineation.

### 2.3 Parallel hierarchical enhancement block

**Figure 4:**
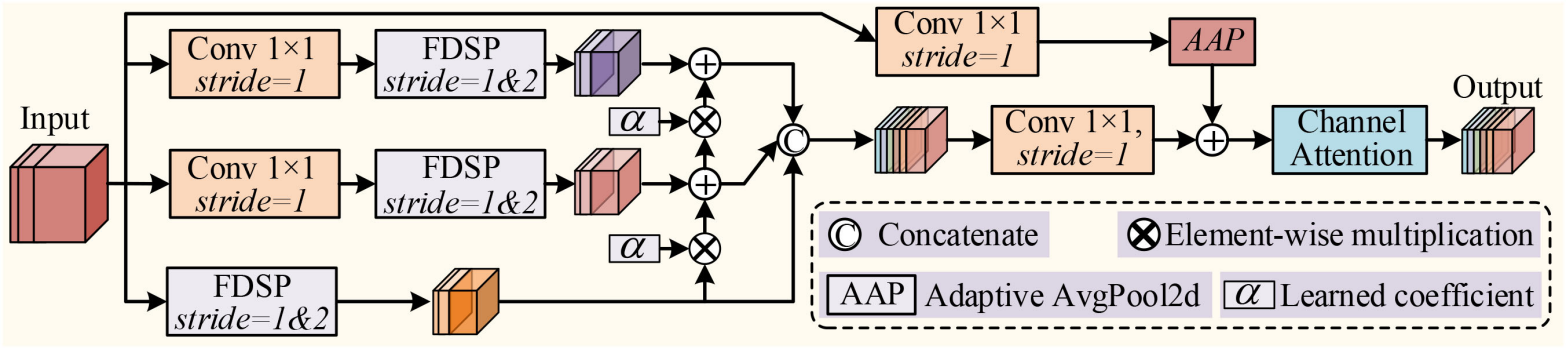
Architecture of the Parallel Hierarchical Enhancement Block (PHEB). The module utilizes parallel FDSP branches for multi-scale extraction, which are subsequently integrated via weak hierarchical interaction and refined through channel-attention recalibration.

To address the significant intra-class variance and boundary ambiguity stemming from the coexistence of bagged and unbagged fruits and their highly irregular distribution within cluster groups, the Parallel Hierarchical Enhancement Block (PHEB) is introduced as an architectural extension of the FDSP module (**Figure 4**). Because bagged and exposed fruits present vastly disparate textural appearances and spatial footprints under unstructured natural lighting, single-receptive-field blocks struggle with localized misclassifications. By employing a parallelized multiscale extraction strategy coupled with weak hierarchical interaction and explicit channel recalibration, this block simultaneously satisfies the rigorous demands for accurate semantic categorization and precise spatial boundary delineation.

Given an input feature map $F_{\text{input}}\in {\mathbb{R}}^{C_{in}\times H\times W}$, the block operates through the following stages.

#### 2.3.1 Parallel multi-scale transformation

To simultaneously capture heterogeneous receptive fields, the input is routed through three parallel FDSP branches. To align channel dimensions for subsequent processing, linear projections are conditionally applied according to Equation 7:

(7) $$X_{1}=F_{\text{input}},\;X_{2}=\phi_{1\times 1}^{\left(2\right)}(F_{\text{input}}),\;X_{3}=\phi_{1\times 1}^{\left(3\right)}(F_{\text{input}})$$

where *φ*_1×1_(·) denotes a 1×1 convolution mapping to an intermediate channel dimension *C_inter_*. Multiscale features are then extracted via branch-specific FDSP modules, as formalized in Equation 8:

(8) $$Y_{i}={\text{FDSP}}^{\left(i\right)}(X_{i};k_{i},r_{i},s_{i}),\;i\in \{1,2,3\}$$

where *k_i_*, *r_i_*, and *s_i_* denote the kernel size, receptive field limit, and stride of the *i*-th branch, respectively.

#### 2.3.2 Adaptive spatial alignment

Because parallel branches may operate under varying strides, an adaptive spatial alignment function A(·) is introduced to dynamically compute a target resolution (*H_t_,W_t_*) based on the minimum dimensions across all branches. Features are strictly aligned via adaptive average pooling or bilinear interpolation, detailed in Equation 9:

(9) $${\bar{Y}}_{i}=A(Y_{i};H_{t},W_{t}),\;i\in \{1,2,3\}$$

#### 2.3.3 Weak hierarchical interaction

To fuse multiscale semantics while preserving intricate spatial details, a weak hierarchical interaction mechanism is applied. Modulated by a learnable scalar parameter *α* (initially 10^−3^), finer-scale features progressively guide coarser representations, established by Equation 10:

(10) $${\tilde{Y}}_{1}={\bar{Y}}_{1},\;{\tilde{Y}}_{2}={\bar{Y}}_{2}+\alpha {\tilde{Y}}_{1},\;{\tilde{Y}}_{3}={\bar{Y}}_{3}+\alpha ({\tilde{Y}}_{1}+{\tilde{Y}}_{2})$$

#### 2.3.4 Feature aggregation and recalibration

Interacted representations are concatenated and fused via a primary transformation $T_{\text{main}}$ comprising 1×1 convolution, Batch Normalization, and ReLU. Concurrently, a residual projection $T_{\text{res}}$ transforms the original input $F_{\text{input}}$, followed by spatial alignment A(·) to ensure dimensional consistency, as computed in Equation 11:

(11) $$F_{\text{main}}=T_{\text{main}}(\text{Concat}({\tilde{Y}}_{1},{\tilde{Y}}_{2},{\tilde{Y}}_{3})),\;F_{\text{res}}=A(T_{\text{res}}(F_{\text{input}});H_{t},W_{t})$$

#### 2.3.5 Final representation

The aggregated context and residual baseline are summed and refined through a global Channel Attention function CA(·) to selectively emphasize informative features, resulting in Equation 12:

(12) $$F_{\text{out}}=CA(F_{\text{main}}+F_{\text{res}})$$

In summary, the PHEB produces highly discriminative representations by integrating parallel multiscale extraction and weak interaction within a compact topology, ensuring robust identification of diverse fruit conditions while maintaining strict spatial alignment.

### 2.4 High-frequency detour state space model

**Figure 5:**
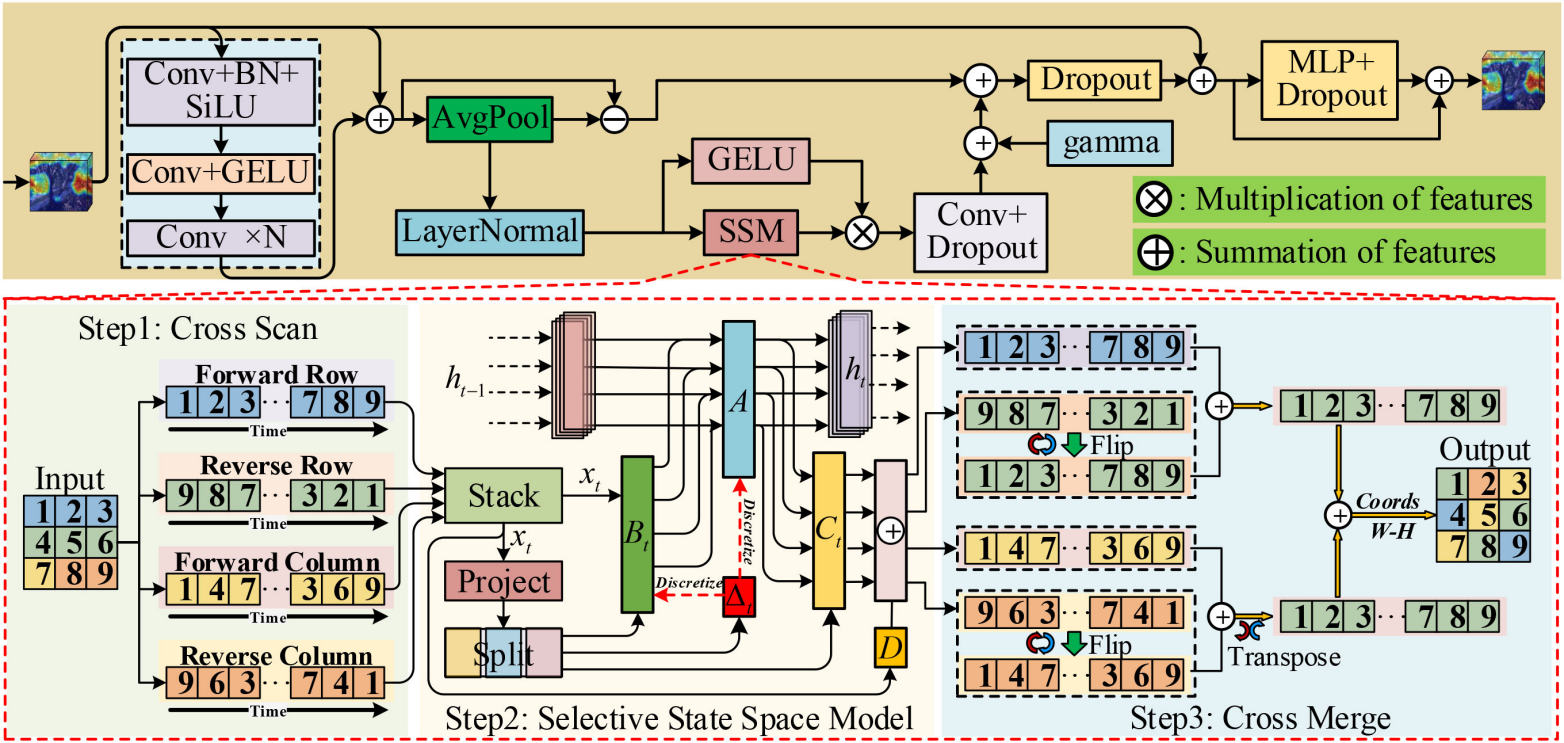
Architecture of the High-frequency Detour State Space Model (HDSSM). The module decomposes the input into a low-frequency semantic base for 2D Selective SSM processing and an unattenuated high-frequency detour to preserve fine-grained spatial details.

Under severe canopy occlusions where targets are heavily fractured by interlaced branches or leaves, resolving global contextual dependency is crucial for re-associating disjointed segments and identifying partial targets. State Space Models (SSMs) effectively capture global dependencies with linear complexity; however, their sequence-based aggregation acts as a low-pass filter, degrading high-frequency spatial details vital for small-target localization and boundary definition under fluctuating orchard illumination. To mitigate this limitation, the High-frequency Detour State Space Model (HDSSM) block is introduced (**Figure 5**). HDSSM delegates holistic semantic modeling to the SSM core while explicitly routing local boundary cues through an unattenuated high-frequency detour.

Given an input tensor $X_{\text{in}}\in {\mathbb{R}}^{C_{\text{in}}\times H\times W}$, a localized non-linear projection maps the representation to a unified hidden dimension alongside a primary residual connection, as shown in Equation 13:

(13) $$X_{1}=F_{\text{local}}(\delta (\text{BN}(f_{1\times 1}(X_{\text{in}}))))+X_{\text{in}},\;X_{1}\in {\mathbb{R}}^{C\times H\times W}$$

To prevent structural degradation, *X*_1_ is decomposed into a low-frequency semantic base and a high-frequency spatial residual. A localized average-pooling filter establishes the semantic baseline, facilitating - high-frequency detour extraction via element-wise subtraction, detailed in Equation 14:

(14) $$X_{\text{low}}=\text{AvgPool}(X_{1}),\;X_{\text{high}}=X_{1}-X_{\text{low}}$$

#### 2.4.1 2D selective state space modeling

To accommodate the non-causal nature of spatial data, a **Cross Scan** operation unfolds red $\text{Norm}(X_{\text{low}})$ into four directional 1D sequences ${\left\{x^{\left(k\right)}\right\}}_{k=1}^{4}$ of length L=H×W. For each sequence, the **Selective State Space Model** dynamically modulates information flow. To achieve input-aware selectivity, the step size ${\text{Δ}}_{t}$ and projection matrices ( $B_{t},C_{t}$) are generated via a joint linear mapping ( $W_{p}^{\left(k\right)}$) of the current input $x_{t}^{\left(k\right)}$, as formulated in Equation 15:

(15) $$\left[{\text{Δ}}_{t}^{\text{proj}},B_{t},C_{t}]=\text{Split}(W_{p}^{\left(k\right)}x_{t}^{\left(k\right)}),\;{\text{Δ}}_{t}=\text{Softplus}({\text{Linear}}_{R\to C}({\text{Δ}}_{t}^{\text{proj}})\right)$$

Continuous-time transition matrices are discretized via the zero-order hold (ZOH) rule: ${\bar{A}}_{t}=\text{exp }({\text{Δ}}_{t}A^{\left(k\right)})$ and ${\bar{B}}_{t}\approx {\text{Δ}}_{t}B_{t}$. The hidden state *h*_*t*_ and sequential $y_{\text{t}}^{\left(k\right)}$ output are recursively updated according to Equation 16:

(16) $$h_{t}={\bar{A}}_{t}h_{t-1}+{\bar{B}}_{t}x_{t}^{\left(k\right)},\;y_{t}^{\left(k\right)}=C_{t}h_{t}+D^{\left(k\right)}x_{t}^{\left(k\right)}$$

where $D^{\left(k\right)}$ functions as a static skip connection.

Modeled sequences ${\left\{y^{\left(k\right)}\right\}}_{k=1}^{4}$ undergo a **Cross Merge** operation to reconstruct the 2D topography. Directional sequences are spatially aligned, summed, and reshaped, then modulated by a gated activation mechanism to yield the global semantic representation *Y*_ssm_.

#### 2.4.2 High-frequency detour and residual aggregation

Unlike standard residual designs that scale the entire feature—inadvertently suppressing weak high-frequency signals—a channel-wise modulation factor $\gamma \in {\mathbb{R}}^{C}$ is applied *strictly* to $Y_{ssm}$. This allows the unattenuated detour $X_{\text{high}}$ to be directly injected, as expressed in Equation 17:

(17) $$Y_{\text{fused}}=X_{\text{high}}+(\gamma ⊙Y_{\text{ssm}})$$

This formulation ensures that pristine local details bypass the contracting state-space transformations. Finally, the fused representation is integrated with *X*_1_ and refined via a feed-forward network, defined by Equation 18:

(18) $$X_{\text{res}}=X_{1}+\text{Dropout}(Y_{\text{fused}}),\;X_{\text{out}}=X_{\text{res}}+\text{Dropout}(F_{\text{mlp}}(\text{Norm}(X_{\text{res}})))$$

In essence, HDSSM establishes a functional dichotomy: dynamic state-space recurrence for global semantics and a parallel detour for high-frequency morphology preservation. The procedure is detailed in Algorithm 1.

Algorithm 1

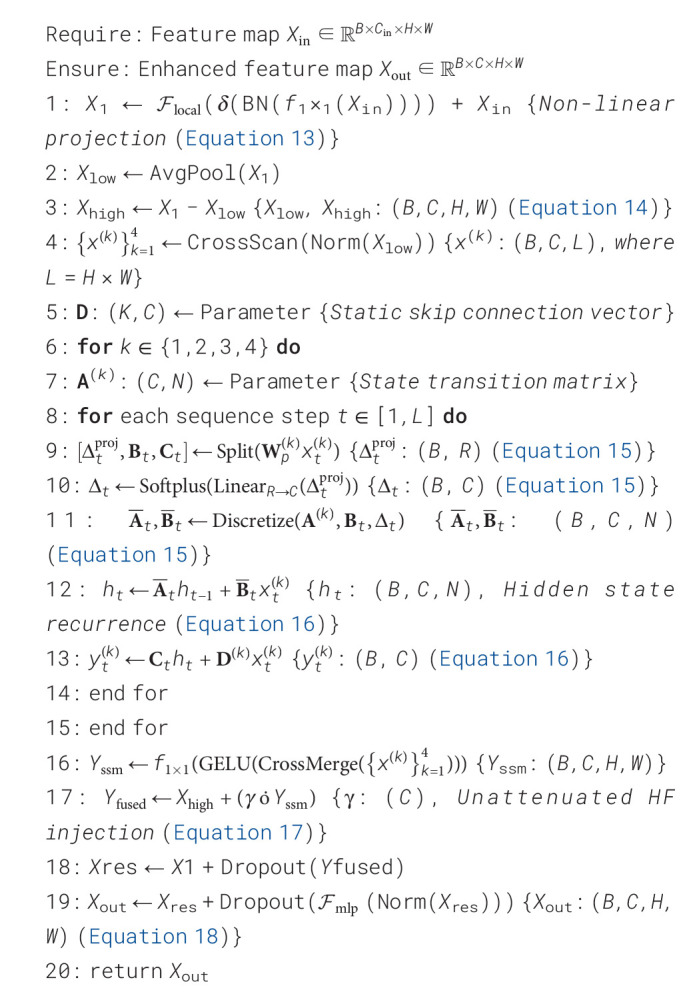

### 2.5 Spatially-refined adaptive fusion module

**Figure 6:**
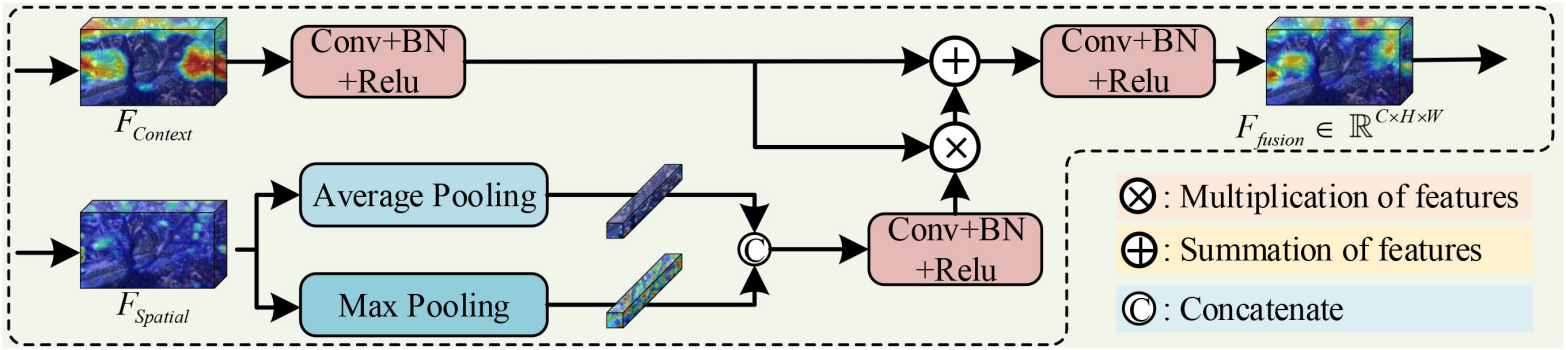
Architecture of the Spatially-Refined Adaptive Fusion (SRAF) module. Spatial attention is derived exclusively from the spatial branch to selectively modulate incoming semantic context, thereby bridging the semantic gap while strictly preserving the morphological boundaries of small targets.

When merging dual-pathway representations in messy background setups, naive element-wise concatenation or addition allows deep semantic feature maps to overwhelm the weak activations of distant or small-scale fruits. The Spatially-Refined Adaptive Fusion (SRAF) module is introduced to bridge the semantic gap between dual pathways without compromising the spatial footprints of small targets (**Figure 6**). Unlike conventional dense fusion, SRAF dynamically filters semantic context prior to spatial injection. The complete stepwise procedure is outlined in Algorithm 2.

Algorithm 2

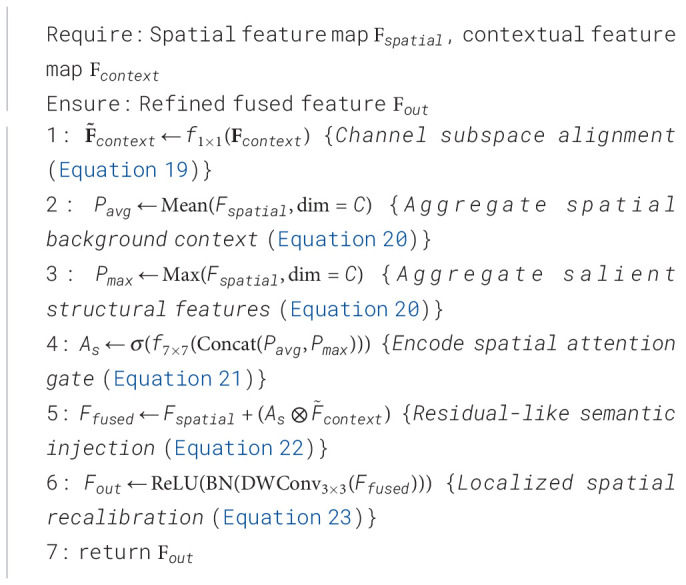

Let $F_{spatial},F_{context}\in {\mathbb{R}}^{C\times H\times W}$ denote features from the spatial and context branches, respectively. To align the channel subspace without lossy compression, $F_{context}$ initially undergoes a bias-free 1×1 linear projection $f_{1\times 1}$, as expressed in Equation 19:

(19) $${\tilde{F}}_{context}=f_{1\times 1}(F_{context})$$

To identify regions requiring semantic reinforcement while safeguarding local details, spatial attention is derived exclusively from the spatial branch. Cross-channel statistics are aggregated via average- and max-pooling to capture background context and salient structures simultaneously, detailed in Equation 20:

(20) $$P_{avg}=\frac{1}{C}\sum_{c=1}^{C}F_{spatial}^{\left(c\right)},\;P_{max}=\underset{c\in \{1,\ldots ,C\}}{\max}F_{spatial}^{\left(c\right)}$$

These descriptors are concatenated and mapped through a 7×7 convolution ( $f_{7\times 7}$), followed by sigmoid normalization (*σ*), generating the spatial attention gate, formulated as Equation 21: $A_{s}\in {\mathbb{R}}^{1\times H\times W}$:

(21) $$A_{s}=\sigma (f_{7\times 7}([P_{avg},P_{max}]))$$

SRAF utilizes **A***_s_* to dynamically modulate aligned contextual representations. This residual-like injection ensures that high-frequency responses intrinsic to $F_{spatial}$ are strictly preserved, as shown in Equation 22:

(22) $$F_{fused}=F_{spatial}+(A_{s}⊗{\tilde{F}}_{context})$$

where ⊗ denotes spatial element-wise multiplication with channel-wise broadcasting. Finally, to alleviate aliasing and enhance boundary continuity, the fused representation undergoes localized spatial recalibration via a 3×3 depthwise convolution ( $D_{3\times 3}$), Batch Normalization ( BN), and ReLU activation ( δ), defined by Equation 23:

(23) $$F_{out}=\delta (\mathrm{BN}(D_{3\times 3}(F_{fused})))$$

By deriving attention exclusively from the spatial pathway, SRAF successfully injects essential semantic context while safeguarding the morphological boundaries of small instances.

### 2.6 Unified detection-segmentation head and objective

To translate fused multiscale representations into instance-level predictions, a unified head bifurcates into parallel streams: classification, bounding-box regression, and dynamic kernel generation. While classification and regression branches compute category probabilities and spatial offsets, the dynamic kernel generator derives instance-specific weights $\theta_{j}$. Concurrently, a mask-feature extractor aggregates multiscale representations with high-resolution cues to construct a shared prototype mask $F^{\text{mask}}$. The final segmentation mask *M_j_* for the *j*-th instance is dynamically assembled, as given by Equation 24:

(24) $$M_{j}=\theta_{j}⊗F^{\text{mask}}$$

where ⊗ denotes dynamic convolution. Predictive streams are jointly optimized via a weighted composite loss, formulated in Equation 25:

(25) $$L=\lambda_{\text{cls}}L_{\text{cls}}+\lambda_{\text{reg}}L_{\text{reg}}+\lambda_{\text{mask}}L_{\text{mask}}$$

Quality Focal Loss (QFL) constitutes $L_{\text{cls}}$ to couple classification and localization confidences, while Generalized IoU (GIoU) formulates $L_{\text{reg}}$ for scale-invariant spatial regression.

For the segmentation objective $L_{\text{mask}}$, standard Dice loss exhibits inherent scale bias, as gradient contributions from varying object sizes disproportionately skew optimization. redThis optimization imbalance is especially pronounced in apple harvesting tasks due to the coexistence of massive close-up fruits and highly diminutive, distant background fruits under irregular tree distributions. The baseline Dice penalty between predicted mask p and ground truth g is defined by Equation 26:

(26) $$L_{\text{dice}}(p,g)=1-\frac{2\sum p\cdot g+\epsilon }{\sum p^{2}+\sum g^{2}+\epsilon }$$

To counteract this field-specific scene scale imbalance, an Area-Stratified Dice (AS-Dice) penalty is formulated to adaptively modulate the baseline penalty in Equation 26 based on the ground-truth instance area $A_{j}$. A scale-adaptive weighting factor $\omega (A_{j})$ is defined as a discrete step function governed by area thresholds *τ*_1_ and *τ*_2_, structured as Equation 27:

(27) $$\omega (A_{j})=\{\begin{array}{ll} \omega_{1}, & A_{j}\leq \tau_{1}\\ \omega_{2}, & \tau_{1}<A_{j}\leq \tau_{2}\\ \omega_{3}, & A_{j}>\tau_{2} \end{array}$$

where hyperparameters $\omega_{1}$, $\omega_{2}$, and $\omega_{3}$ explicitly calibrate learning signals across spatial extents. Empirically, thresholds are set to $\tau_{1}=3000$ and $\tau_{2}=8000$ pixels, with weights $\omega_{1}=1.0$, $\;\omega_{2}=0.8$, and $\omega_{3}=0.9$. By assigning the highest weight to the smallest scale ( $A_{j}\leq \tau_{1}$), AS-Dice amplifies the inherently weak gradients of diminutive targets, preventing morphological detail suppression during optimization. The final mask loss over $N_{\text{inst}}$ instances is computed according to Equation 28:

(28) $$L_{\text{mask}}=\frac{1}{N_{inst}}\sum_{j=1}^{N_{\text{inst}}}\omega (A_{j})\cdot L_{\text{dice}}(p_{j},g_{j})$$

By normalizing gradient propagation across variable scales, AS-Dice stabilizes the optimization trajectory and guarantees consistent boundary delineation for both small and large instances.

## 3 Apple instance segmentation dataset

### 3.1 Data collection

The apple instance segmentation dataset was collected in a commercial orchard in Liquan County, Shaanxi Province, China, during the 2024 harvest season (September–October). To simulate a robotic manipulator’s workspace, an Intel RealSense D435i camera was positioned 50–80 cm from the fruiting canopy.

To capture the high intra-class variance typical of modern cultivation, the dataset incorporates a diverse mixture of bagged and unbagged mature apples. Images were acquired under various meteorological conditions and times of day to ensure variability in illumination and shadowing. This strategy deliberately introduces real-world visual degradations, such as dense foliage occlusion and severe specular reflections from plastic bagging.

**Figure 7:**
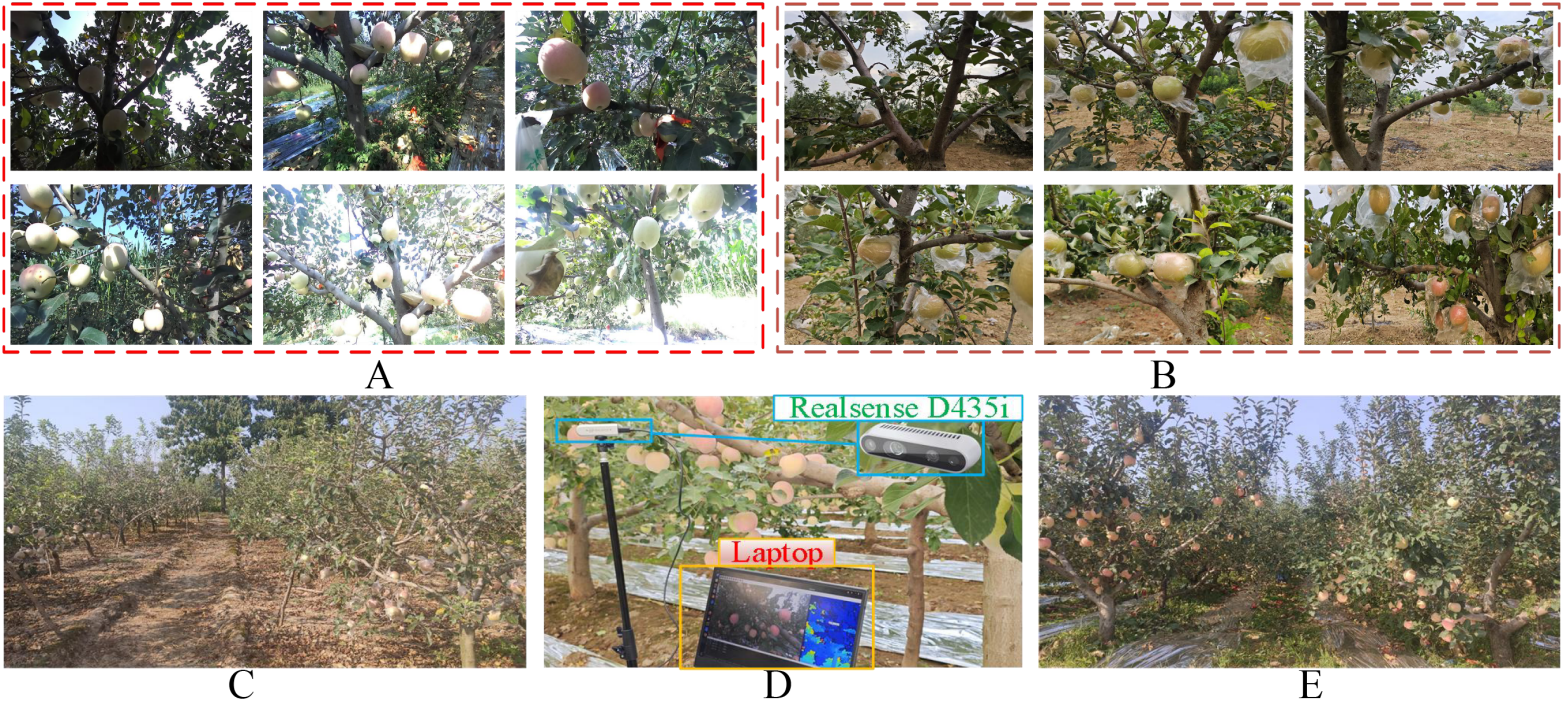
Overview of data collection and representative samples. **(A)** Unbagged apples; **(B)** Bagged apples; **(C)** Bagged apple orchard scene; **(D)** Recording equipment setup (Intel RealSense D435i); **(E)** Unbagged apple orchard scene.

As illustrated in **Figure 7**, these environmental variations are captured alongside the hardware configuration, showcasing representative samples of diverse orchard scenes. After filtering invalid captures, a total of 2,259 high-quality images were retained, containing 46,511 annotated instances with an average of 20.59 instances per image. Ground-truth masks were initially generated using the Segment Anything Model (SAM) (Kirillov et al., 2023) and subsequently refined manually to ensure strict boundary precision. To provide a comprehensive architectural profile of the dataset, the detailed statistical distributions—including bagged/unbagged categories, object scales (small, medium, large), and inter-object occlusion characteristics across different subsets—are summarized in **Table 1**. Finally, the dataset was partitioned into training, validation, and testing subsets, comprising 1,329, 443, and 487 images, respectively.

**Table 1:** Detailed statistical attributes and subset distributions of the collected apple dataset.

Dataset property/metric	Train	Val	Test	Total
Total Number of Images	1329	443	487	2259
Total Annotated Instances	27701	8764	10046	46511
Average Instances per Image	20.84	19.78	20.63	20.59
Bagged Apples Instances Count	6344	2174	3961	12479
Unbagged Apples Instances Count	21357	6590	6085	34032
Small Objects (< 322 px)	6004	1844	2707	10555
Medium Objects (322–962 px)	17128	5423	5774	28325
Large Objects (> 962 px)	4569	1497	1565	7631
Inter-Object Occluded Instances	5796	1740	1727	9263
Occlusion Ratio (%)	20.92%	19.85%	17.19%	19.92%

### 3.2 Dynamic data augmentation and training strategy

A two-stage dynamic data augmentation paradigm is implemented to mitigate overfitting and enhance generalization across unstructured orchard conditions. Departing from static transformations, this pipeline modulates augmentation intensity across training phases to balance data diversity with optimal convergence.

During the primary phase, robust spatial and photometric perturbations are applied. Mosaic augmentation expands contextual variability, simulating dense, overlapping fruit distributions. This synergizes with large-scale random resizing (0.1–2.0×), spatial cropping, horizontal flipping, and HSV jittering to synthesize the complex illumination and scale variations inherent to real-world canopies.

In the final fine-tuning phase (spanning epochs *E_m_* to *E_max_*), strong distortions including Mosaic and arbitrary cropping are deactivated. The pipeline transitions to a lightweight configuration—comprising constrained resizing (0.8–1.2×) and standard flipping—to align the augmented feature space with the authentic data distribution, thereby stabilizing terminal convergence. **Table 2** summarizes this schedule; specific epoch boundaries (e.g., *E_w_*, *E_m_*, *E_max_*) are detailed in Sec. 4.

**Table 2:** Two-stage dynamic training schedule and stage-wise augmentation strategies.

Stage (Epochs)	LR schedule	Augmentation strategy
Warm-up (1 → *E*_*w*_)	Linear (0.25 → 1.0×)	Strong: Mosaic, resize, crop, HSV, flip
Main (*E*_*w*_ → *E*_*m*_)	Cosine (*η*_*min*_ = *η*_*base*_/30)	Strong: Mosaic, resize, crop, HSV, flip
Fine-tune (*E*_*m*_ → *E*_*max*_)	Cosine (terminal)	Light: Resize (0.8–1.2×), HSV, flip (*Mosaic off*)

## 4 Experiment

### 4.1 Implementation details

To ensure experimental fairness, all models are trained under identical hardware conditions. Comparative evaluations include seven baseline networks implemented via the MMDetection v3.3.0 framework (Chen et al., 2019). Furthermore, state-of-the-art YOLO variants are benchmarked using the Ultralytics v8.3.163 environment. While the hardware remains consistent across all trials, the software stack is tailored to each specific framework (**Table 3**).

**Table 3:** Hardware and software specifications across baseline and optimization environments.

Category	MMDetection environment	YOLO environment
CPU	Intel Xeon Silver 4214	
Memory	64 GB (4×16 GB) 2666 MHz DDR4 ECC	
GPU	NVIDIA RTX 3090 Ti (single)	
DL Framework	MMDetection v3.3.0	Ultralytics v8.3.163
Python	3.8.19	3.10.18
PyTorch/CUDA	2.0.1/11.8	2.0.0/11.8
TorchVision	0.15.2	0.15.1
MMCV/MMEngine	2.0.1/0.10.5	N/A
NumPy	1.24.4	1.24.0

The LRD-Inst framework executes joint object detection and instance segmentation via a multi-task objective incorporating classification, regression, and scale-aware mask supervision. During inference, predictions are filtered using non-maximum suppression (IoU threshold = 0.65) and a 0.05 confidence threshold.

Training spans 720 epochs with a batch size of 6 via the AdamW optimizer (*β* = (0.9, 0.999), weight decay = 0.05) and gradient clipping (max norm = 1.0). The base learning rate of 0.004 follows a three-stage schedule: a 2-epoch linear warm-up, a cosine annealing decay, and a simplified augmentation fine-tuning switch for the final 80 epochs. An exponential moving average (EMA) with a 0.0002 momentum stabilizes the trajectory.

### 4.2 Quantitative assessment criteria

To rigorously quantify the segmentation fidelity and hardware deployability of LRD-Inst, evaluation follows standard COCO criteria. Foundational metrics, Precision (*P*) and Recall (*R*), are formulated via confusion matrix components, as computed in Equation 29:

(29) $$P=\frac{TP}{TP+FP},\;R=\frac{TP}{TP+FN},$$

where *TP*, *FP*, and *FN* represent true positives, false positives, and false negatives, respectively. Segmentation accuracy is evaluated using Average Precision (AP) and Average Recall (AR), averaged across Intersection over Union (IoU) thresholds from 0.50 to 0.95. To assess boundary adherence, AP_50_, AP_75_, and AP_95_ are reported, reflecting performance under progressively stringent overlap criteria. Robustness against scale imbalance is benchmarked by stratifying metrics into small (AP*_S_*, AR*_S_*), medium (AP*_M_*, AR*_M_*), and large (AP*_L_*, AR*_L_*) categories based on pixel area.

Finally, architectural complexity and inference efficiency are quantified via three metrics: Params (learnable parameters), GFLOPs (floating-point operations per inference), and FPS (frames processed per second) to validate real-time feasibility on edge devices.

### 4.3 Benchmark results

To evaluate the segmentation efficacy of LRD-Inst, comparative experiments were conducted on the self-constructed mixed-apple dataset. The framework is benchmarked against a diverse spectrum of state-of-the-art architectures, encompassing classical two-stage paradigms (Mask R-CNN), dynamic-kernel methods (CondInst, SOLOv2, PointRend), and recent lightweight one-stage models (RTMDet and YOLOv8–YOLOv26 families). To emphasize the boundary delineation capabilities essential for agricultural deployment, evaluation focuses exclusively on instance segmentation metrics.

**Figure 8:**
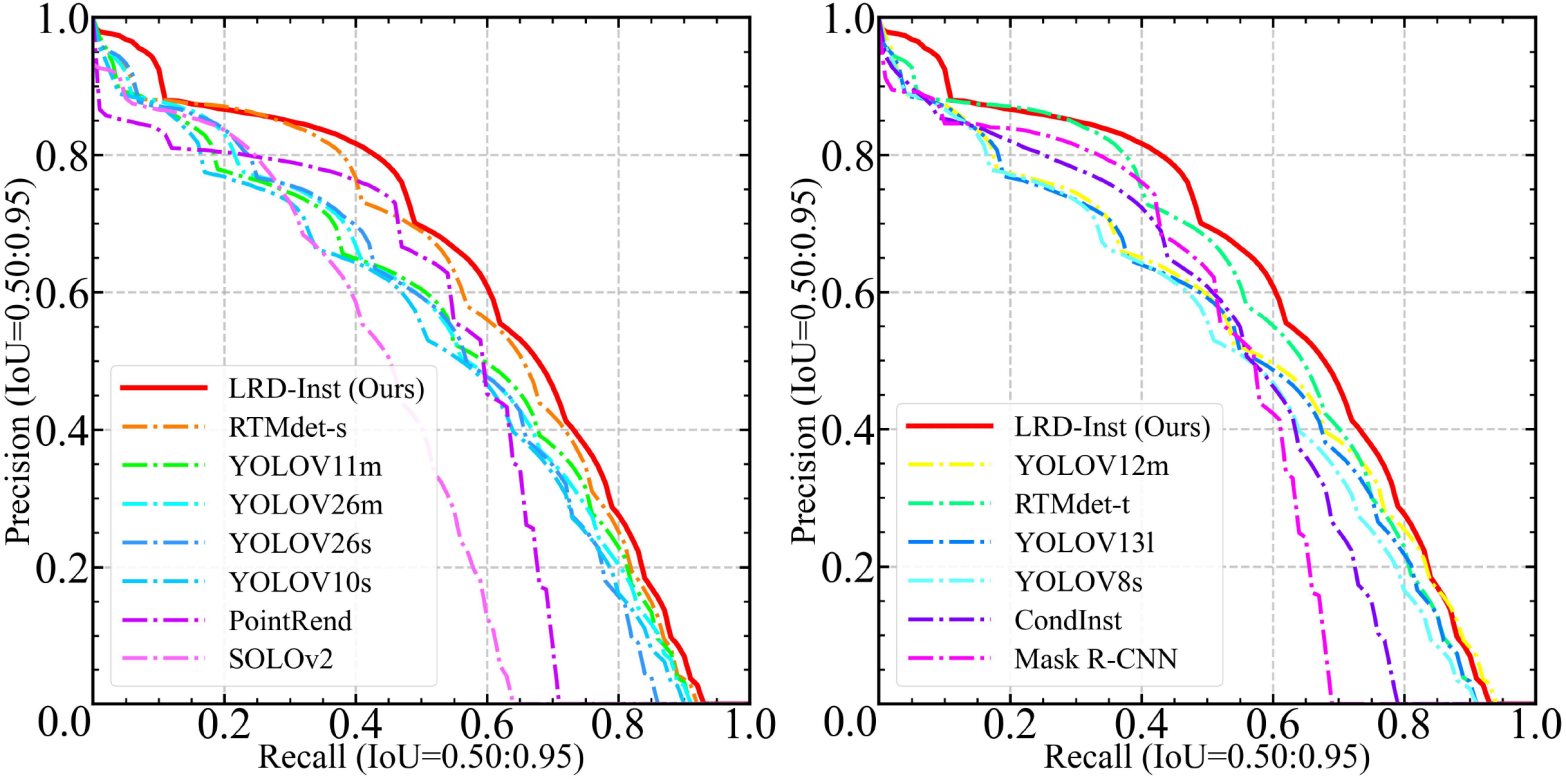
Precision-Recall (PR) curves evaluated at IoU=0.50:0.95. To ensure visual clarity among the 15 evaluated models, the comparisons are divided into two subplots: LRD-Inst (Ours) against the first 7 baselines (left) and the remaining 7 variants (right).

As detailed in **Table 4**, LRD-Inst establishes a comprehensive state-of-the-art for apple targets on the independent test set, achieving a primary AP of 0.568. **Figure 8** plots the Precision–Recall (PR) curves across the primary IoU range (0.50:0.95), demonstrating that LRD-Inst maintains a superior precision–recall envelope that outpaces dynamic-kernel CondInst (0.467, + 21.6%), lightweight RTMDet-s (0.534, + 6.4%), and the highly optimized YOLO series (e.g., YOLOv9c, YOLOv26m). This consistent lead confirms the architectural superiority of the spatially-refined dual-branch topology.

**Table 4:** Quantitative comparison of instance segmentation performance on the independent test set.

Model	AP	AP_50_	AP_75_	AP_95_	AP_S_	AP_M_	AP_L_	A_R_	AR_S_	AR_M_	AR_L_
PointRend (Kirillov et al., 2020)	0.458	0.646	0.510	0.037	0.190	0.517	0.693	0.543	0.310	0.600	0.736
SOLOv2 (Wang et al., 2020)	0.372	0.581	0.394	0.018	0.064	0.498	0.727	0.435	0.131	0.498	0.729
CondInst (Tian et al., 2020)	0.467	0.681	0.516	0.035	0.197	0.535	0.726	0.569	0.339	0.625	0.764
Mask R-CNN (He et al., 2017)	0.451	0.635	0.504	0.043	0.200	0.497	0.661	0.517	0.294	0.571	0.705
YOLOv8s (Jocher et al., 2023)	0.502	0.787	0.550	0.022	0.214	0.547	0.738	0.588	0.412	0.622	0.766
YOLOv9c (Wang et al., 2024b)	0.524	0.804	0.580	0.024	0.242	0.562	0.748	0.608	0.450	0.636	0.778
YOLOv10s (Wang et al., 2024a)	0.500	0.781	0.547	0.022	0.214	0.542	0.735	0.585	0.413	0.617	0.765
YOLOv11m (Khanam and Hussain, 2024)	0.528	0.803	0.588	0.028	0.250	0.570	0.747	0.609	0.445	0.641	0.773
YOLOv12m (Tian et al., 2025)	0.532	0.824	0.585	0.025	0.242	0.576	0.760	0.620	0.447	0.655	0.791
YOLOv13l (Lei et al., 2025)	0.518	0.793	0.580	0.022	0.226	0.561	0.740	0.606	0.442	0.638	0.770
YOLOv26s (Sapkota et al., 2025)	0.518	0.758	0.574	0.038	0.213	0.567	0.757	0.598	0.395	0.642	0.790
YOLOv26m (Sapkota et al., 2025)	0.526	0.789	0.574	0.045	0.230	0.571	0.760	0.627	0.465	0.659	0.793
RTMDet-tiny Lyu et al. (2022)	0.526	0.796	0.579	0.027	0.219	0.573	0.772	0.611	0.401	0.655	0.815
RTMDet-s Lyu et al. (2022)	0.534	0.801	0.588	0.036	0.225	0.583	0.781	0.614	0.401	0.658	0.819
LRD-Inst (Ours)	0.568	0.815	0.618	0.077	0.280	0.615	0.788	0.653	0.478	0.692	0.815

“AP” and “AR” denote Average Precision and Average Recall across varying IoU thresholds and object scales.

**Figure 9:**
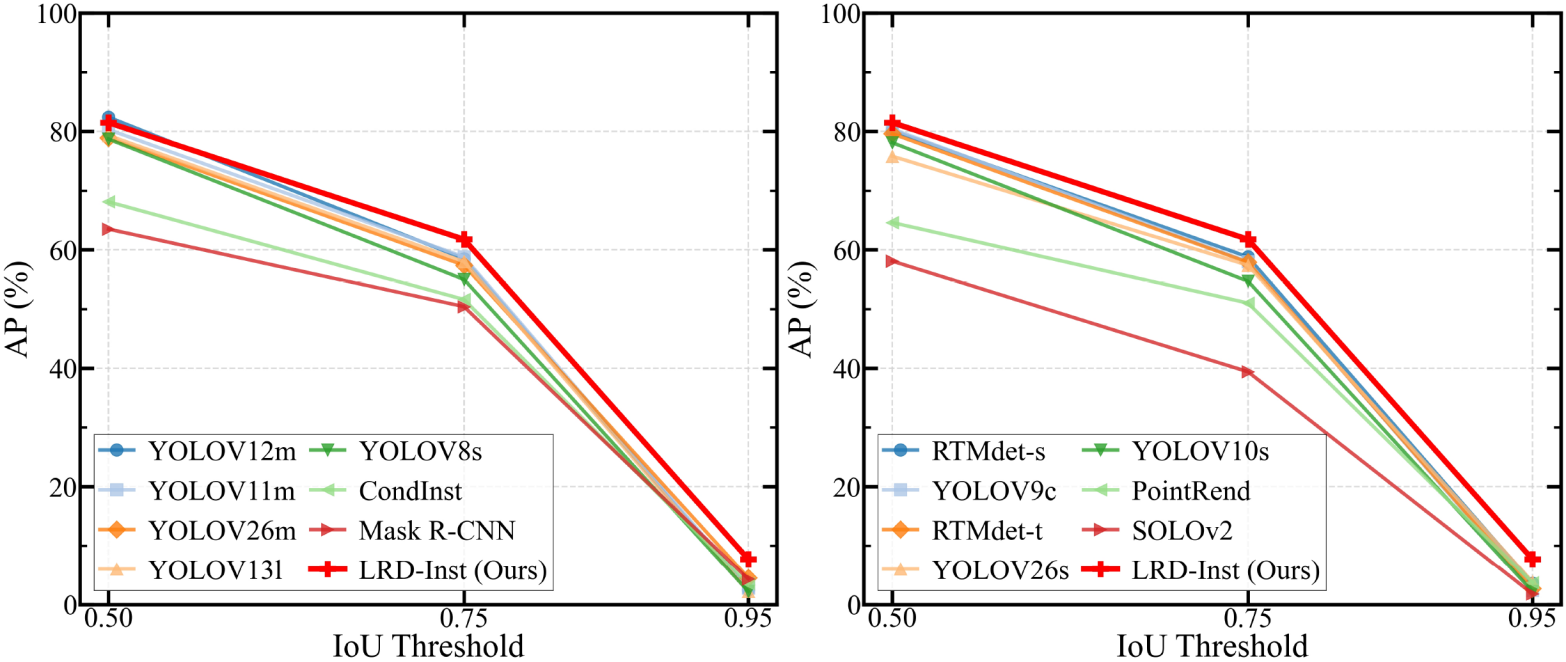
Multi-threshold AP scatterand line plots (AP_50_, AP_75_, AP_95_) illustrating model performance decay under increasingly stringent boundary criteria. The results are separated into two subplots for clarity.

The most distinguishing advantage of LRD-Inst emerges under stringent IoU evaluation criteria. Although trailing slightly behind YOLOv12m at the loose AP_50_ threshold, LRD-Inst demonstrates exceptional precision with AP_75_ = 0.618 and a remarkable AP_95_ = 0.077. The multi-threshold performance decay (AP_50_ → AP_95_) visualized in **Figure 9** confirms that LRD-Inst degrades significantly slower than its counterparts. Notably, the AP_95_ score is more than double that of established models like RTMDet-s (0.036). This profound resilience validates the proposed high-frequency detour and AS-Dice loss, which explicitly penalize boundary deviations and preserve morphology despite severe specular reflections from bagged fruits.

Scale imbalance remains a persistent bottleneck in dense canopy vision. As shown in **Table 4**, LRD-Inst resolves this challenge, registering robust performance across all dimensions (AP*_M_* = 0.615, AP*_L_* = 0.788). Crucially, for small and heavily occluded instances, LRD-Inst achieves AP*_S_* = 0.280 and AR*_S_* = 0.478, unequivocally outperforming CondInst (0.197/0.339) and RTMDet-s (0.225/0.401).

To examine model robustness against specific unstructured field challenges, a fine-grained sub-scenario error analysis was executed for apple targets on the independent test set. **Table 5** presents the segment AP scores under isolated operational conditions, including protective bagging, dense cluster occlusions, and their respective counter-scenarios.

**Table 5:** Sub-scenario error analysis (AP_segm_) under specific operational profiles on the independent test set, focusing on tactile surface variations and overlapping cluster masking.

Model	Bagged	Unbagged	Occluded	Non-occluded
PointRend (Kirillov et al., 2020)	0.384	0.511	0.473	0.495
Mask R-CNN (He et al., 2017)	0.354	0.517	0.433	0.485
CondInst (Tian et al., 2020)	0.369	0.532	0.483	0.515
YOLOv8s (Jocher et al., 2023)	0.417	0.561	0.445	0.551
YOLOv9c (Wang et al., 2024b)	0.446	0.576	0.565	0.575
YOLOv10s (Wang et al., 2024a)	0.412	0.561	0.540	0.553
YOLOv11m (Khanam and Hussain, 2024)	0.446	0.583	0.569	0.575
YOLOv12m (Tian et al., 2025)	0.459	0.582	0.575	0.584
YOLOv13l (Lei et al., 2025)	0.433	0.578	0.563	0.574
YOLOv26s (Sapkota et al., 2025)	0.428	0.581	0.555	0.565
YOLOv26m (Sapkota et al., 2025)	0.454	0.575	0.571	0.575
RTMDet-tiny Lyu et al. (2022)	0.444	0.584	0.563	0.573
RTMDet-s Lyu et al. (2022)	0.447	0.590	0.568	0.581
LRD-Inst (Ours)	0.486	0.627	0.610	0.614

The empirical profiles in **Table 5** confirm that protective bagging and cluster occlusions remain pervasive performance bottlenecks across all architectures. For instance, the baseline RTMDet-s exhibits a 0.143 AP accuracy drop when transitioning to reflective bagged surfaces, underscoring the severe degradation induced by plastic film distortions. Similarly, dense target overlaps compromise mask coherence across standard YOLO networks due to local geometric masking. Conversely, LRD-Inst demonstrates pronounced resilience against these environmental constraints, securing top-tier scores under both bagged (0.486 AP) and occluded (0.610 AP) configurations. This cross-scenario stability validates that isolating high-frequency boundary cues prevents morphological erosion, while global state-space modeling sustains target integrity under severe spatial overlap, maintaining a robust precision balance where traditional dense attention mechanisms degrade.

**Figure 10:**
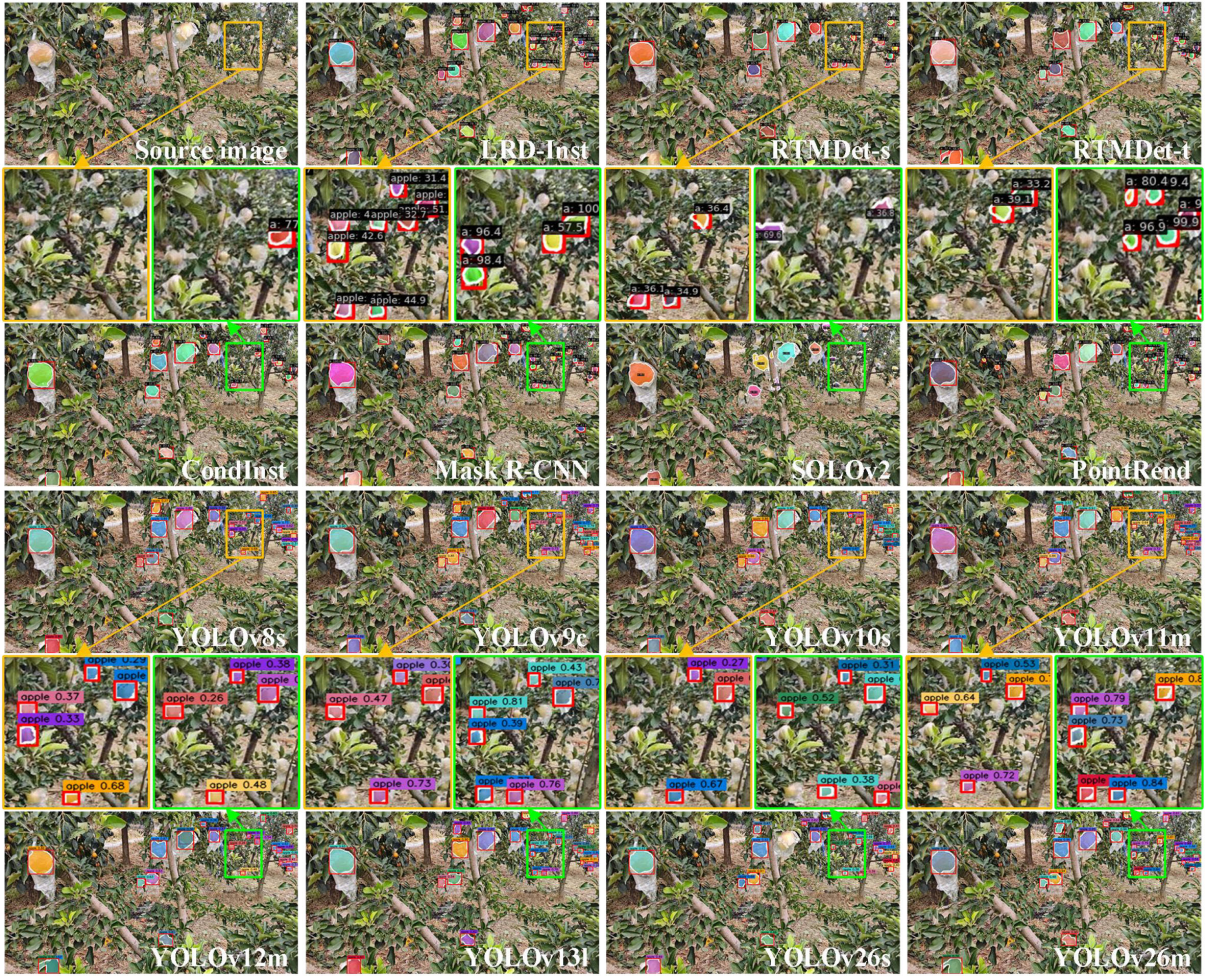
Qualitative comparison of instance segmentation performance on bagged apples. LRD-Inst demonstrates robust recognition and cohesive mask generation despite severe specular reflections and plastic film distortions.

**Figure 11:**
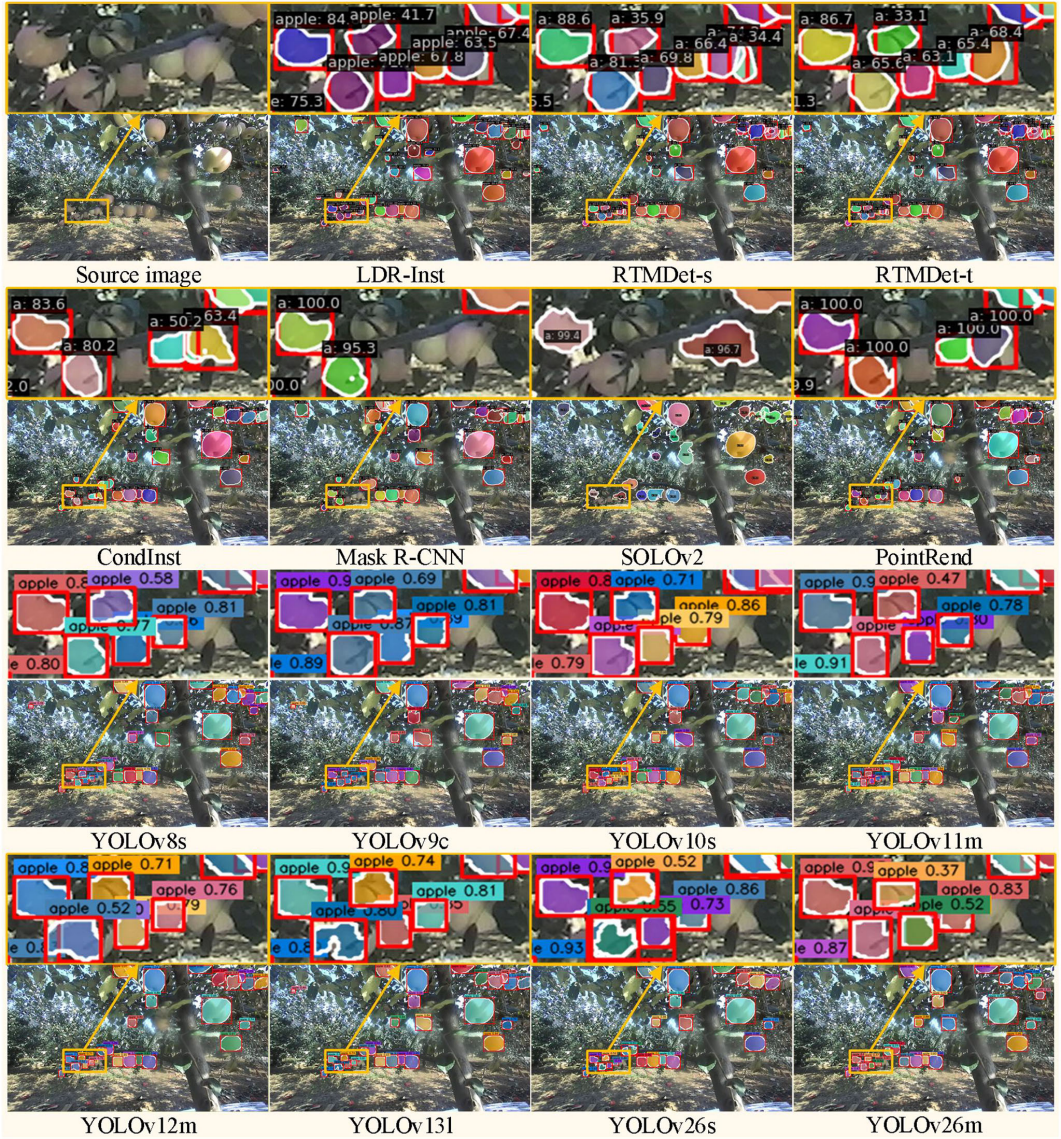
Qualitative comparison of instance segmentation performance on unbagged apples. LRD-Inst maintains highly precise boundary delineations under natural illumination and dense canopy occlusions.

Visual qualitative comparisons further elucidate the model’s superiority under challenging physical conditions. **Figures 10** and **11** contrast the segmentation masks generated for bagged and unbagged apples, respectively. These visualizations confirm that LRD-Inst consistently produces the most cohesive masks and sharpest boundaries at the micro level, successfully overcoming severe specular reflections and heavy foliage occlusions. Its unparalleled capability to delineate boundaries under extreme intra-class variance establishes LRD-Inst as an optimal solution for agricultural robotic operations.

To verify the statistical validity of the performance advancements and eliminate optimization variance, localized replication trials were executed. **Table 6** presents the mean metrics and standard deviations (Mean ± SD) compiled across five fully synchronized independent runs for the top-performing baselines and LRD-Inst.

**Table 6:** Statistical reproducibility analysis across five independent trials evaluated on the independent test set. Results are formatted as Mean ± SD.

Model	AP	AP_50_	AP_75_	A_R_	AR_S_	AR_L_
YOLOv12m (Tian et al., 2025)	0.532 ± 0.004	0.824 ± 0.004	0.585 ± 0.005	0.622 ± 0.004	0.452 ± 0.005	0.791 ± 0.005
RTMDet-s Lyu et al. (2022)	0.534 ± 0.004	0.801 ± 0.007	0.588 ± 0.004	0.614 ± 0.003	0.401 ± 0.002	0.819 ± 0.003
LRD-Inst (Ours)	0.568 ± 0.002	0.815 ± 0.002	0.618 ± 0.002	0.653 ± 0.001	0.478 ± 0.005	0.815 ± 0.004

The rigorous five-run analysis in **Table 6** confirms that the accuracy gains achieved by LRD-Inst are highly stable and reproducible. Notably, LRD-Inst maintains an exceptionally narrow variance profile (± 0.002 AP), signifying a tightly converged optimization trajectory. Even under the strictest configuration, the minimum performance bound of LRD-Inst (0.566 AP) outpaces the maximum bound of RTMDet-s (0.538 AP), completely isolating their respective confidence intervals. This clear separation demonstrates that the structural optimization provided by the frequency-decoupled framework yields statistically significant enhancements rather than stochastic fluctuations, ensuring reliable boundary parsing for field robotics.

### 4.4 Model complexity and inference efficiency

To assess deployability on mobile agricultural robots, architectural complexity and inference speed were benchmarked on an MSI P6000 industrial computer equipped with an NVIDIA RTX 3060 GPU. As shown in **Table 7**, LRD-Inst exhibits an exceptionally compact topology, requiring merely 9.12 GFLOPs and 3.43 M parameters. Compared to heavy paradigms like Mask R-CNN (261.00 GFLOPs, 44.40 M), this represents a reduction of over 96% in computation and 92% in parameter footprint. Furthermore, relative to RTMDet-tiny, LRD-Inst reduces GFLOPs by 23.2% and parameters by 38.9%.

**Table 7:** Quantitative evaluation of computational complexity and inference efficiency under unified hardware constraints.

Model	FLOPs (G)	Params (M)	FPS
PointRend (Kirillov et al., 2020)	206.00	56.16	13.5
SOLOv2 (Wang et al., 2020)	268.00	32.17	13.3
CondInst (Tian et al., 2020)	337.00	33.90	14.7
Mask R-CNN (He et al., 2017)	261.00	44.40	15.1
YOLOv8s (Jocher et al., 2023)	42.60	11.80	112.4
YOLOv9c (Wang et al., 2024b)	159.40	27.90	64.0
YOLOv10s (Wang et al., 2024a)	21.60	7.20	126.5
YOLOv11m (Khanam and Hussain, 2024)	123.30	22.40	93.4
YOLOv12m (Tian et al., 2025)	67.50	20.20	60.2
YOLOv13l (Lei et al., 2025)	88.40	27.60	30.0
YOLOv26s (Sapkota et al., 2025)	34.20	10.40	89.7
YOLOv26m (Sapkota et al., 2025)	121.50	23.60	74.9
RTMDet-tiny Lyu et al. (2022)	11.87	5.61	53.8
RTMDet-s Lyu et al. (2022)	21.50	10.18	38.1
LRD-Inst (Ours)	9.12	3.43	45.4

During inference, LRD-Inst achieves 45.4 FPS, exceeding the 30 FPS real-time requirement for continuous robotic harvesting. While small-scale models like YOLOv26s execute faster, they exhibit significant degradation in segmentation fidelity and small-target localization. Consequently, LRD-Inst establishes an optimal equilibrium, securing state-of-the-art mask precision while strictly adhering to the hardware constraints of onboard industrial environments.

### 4.5 Convergence analysis

**Figure 12:**
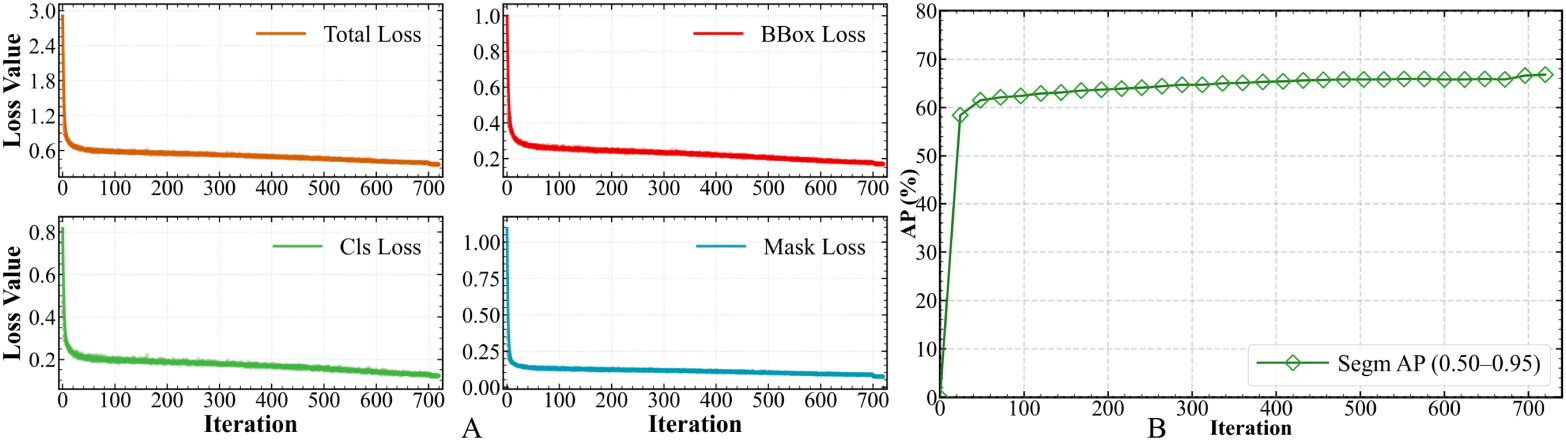
Convergence dynamics of LRD-Inst over 720 epochs. **(A)** Evolution of Total, BBox, Classification, and Mask losses in a four-grid layout. **(B)** Corresponding progression of the AP (0.50:0.95) metric, highlighting rapid initial convergence and terminal optimization stability.

To evaluate the training stability and optimization dynamics of LRD-Inst, **Figure 12** visualizes the multi-task loss descent (**Figure 12A**) and the progressive enhancement of segmentation accuracy (**Figure 12B**) over 720 epochs. Although quantitative benchmarking focuses exclusively on mask segmentation, the unified head of LRD-Inst utilizes bounding-box (BBox) regression for joint optimization during training. Consequently, the dynamics for all predictive streams—encompassing Total, Classification (Cls), BBox, and Mask losses—are compiled simultaneously.

As illustrated in **Figure 12A**, the Total Loss and its sub-components exhibit a steep initial descent followed by a smooth, monotonic reduction. Notably, a synchronized drop occurs across all loss curves around epoch 640, reflecting the transition from cosine annealing to the constant fine-tuning phase (epochs 640–720). This strategic shift facilitates localized parameter refinement, driving a final phase of stringent boundary optimization.

Concurrently, **Figure 12B** demonstrates that the primary metric, AP (0.50:0.95) evaluated on the validation set, mirrors these loss dynamics. The validation AP value surges rapidly during early epochs, achieves steady mid-stage generalization gains, and stabilizes at its peak following the terminal learning-rate transition. This synchronized behavior—well-behaved loss descent coupled with monotonic AP ascent—confirms the convergence robustness and structural stability of the LRD-Inst architecture.

### 4.6 Ablation study

To comprehensively quantify the individual and collective architectural contributions of the proposed modules, ablation experiments were conducted by integrating the Area-Stratified Dice (AS-Dice) loss, the Spatially-Refined Adaptive Fusion (SRAF), and the High-frequency Detour State Space Model (HDSSM). As summarized in **Table 8**, the baseline architecture devoid of all three components achieves a primary AP of 0.554. Substituting the standard Dice loss with the proposed AS-Dice loss improves the accuracy to 0.556 AP. This enhancement is driven by the area-sensitive reweighting mechanism, which prevents morphologically dominant objects from monopolizing optimization, thereby lifting the small-object metric (AP*_S_*) from 0.238 to 0.251.

**Table 8:** Comprehensive ablation study of core components and optimization loss.

Modules/loss			Segmentation accuracy								Complexity	
HDSSM	SRAF	AS-Dice	AP	AP_50_	AP_75_	AP_95_	AP*_S_*	AP*_M_*	AP*_L_*	AR	FLOPs (G)	Params (M)
×	×	×	0.554	0.810	0.602	0.066	0.238	0.606	0.789	0.636	8.63	2.58
×	×	✓	0.556	0.807	0.603	0.066	0.251	0.608	0.786	0.640	8.63	2.58
×	✓	✓	0.559	0.810	0.609	0.068	0.254	0.609	0.796	0.644	8.66	2.63
✓	✓	✓	**0.568**	**0.815**	**0.618**	**0.077**	**0.280**	**0.615**	**0.788**	**0.653**	**9.12**	**3.44**

Performance is evaluated via instance segmentation metrics, highlighting accuracy gains across varying object scales and strict IoU thresholds without introducing redundant inference complexity. Bold values indicate the non-ablated experimental groups.

Further integrating the SRAF module alongside the AS-Dice loss elevates performance to 0.559 AP, validating the efficacy of cross-branch spatial-semantic adaptive feature refinement. Finally, joint deployment of all three components within the full LRD-Inst framework establishes a peak performance of 0.568 AP. These results solidify the critical role of the proposed topology in high-fidelity boundary delineation and scale-invariant perception in complex orchard environments.

Notably, at the most stringent evaluation threshold, the complete LRD-Inst framework increases AP_95_ from 0.066 to 0.077. The impact is particularly profound for diminutive targets, where the baseline registers an AP*_S_* of 0.238, whereas the fully integrated framework surges this metric to 0.280—representing a substantial absolute gain of 4.2%. Crucially, this performance leap is achieved with a highly optimized computational footprint. The transition from the baseline to the full LRD-Inst framework incurs an increase of only 0.49 GFLOPs and 0.86 M parameters. This minimal overhead confirms that the structural allocation design of HDSSM and SRAF, coupled with training-phase optimization of AS-Dice loss, preserves the superior edge-device deployability required for real-time agricultural robotics.

#### 4.6.1 Component-level computational complexity

To clarify the precise computational burden of individual components whose complete removal is architecturally prohibitive, an isolated complexity analysis was executed. **Table 9** reports the independent parameter counts and floating-point operations (FLOPs) contributed by a single forward pass of each module under a standardized intermediate feature tensor $F\in {\mathbb{R}}^{6\times 96\times 80\times 80}$.

**Table 9:** Component-level complexity breakdown under intermediate feature tensor $F\in {\mathbb{R}}^{6\times 96\times 80\times 80}$.

Component	FLOPs (G)	Ratio (%)	Params (M)	Ratio (%)
FDSP	0.043	0.47	0.007	0.20
PHEB	0.367	4.02	0.068	1.98
SRAF	0.011	0.12	0.017	0.49
HDSSM	0.460	5.04	0.811	23.58

Ratios indicate the percentage contribution relative to the complete LRD-Inst framework.

The empirical breakdown demonstrates the high efficiency of the proposed components relative to the total model profile (9.12 GFLOPs/3.44 M parameters). Specifically, FDSP and SRAF operate under an exceptionally compact mathematical envelope, requiring merely 0.043 GFLOPs (0.47%) and 0.011 GFLOPs (0.12%) of the total computational budget, respectively. Similarly, their parameter footprints represent minimal fractions of the complete network (0.20% for FDSP and 0.49% for SRAF). While PHEB and the contextual HDSSM exhibit higher relative overheads due to multi-scale parallel extractions and state-space scanning matrices, their individual footprints remain strictly constrained within lightweight limits, effectively averting hardware-level computational overloading during mobile edge deployment.

#### 4.6.2 Comparison of contextual modeling configurations

To further justify the necessity of utilizing the state-space formulation within the contextual pathway, the proposed HDSSM module was rigorously benchmarked against alternative sequence-modeling and global-attention mechanisms under identical parameter budgets. Specifically, the contextual branch of LRD-Inst was configured using standard multi-head self-attention (MHSA) (Dosovitskiy et al., 2020), Swin Transformer blocks (Liu et al., 2021), and the generic VMamba operator (Liu et al., 2024b), keeping all other architectural components and optimization objectives constant.

Quantitative comparisons in **Table 10** confirm that the HDSSM configuration delivers favorable tradeoffs between segmentation accuracy and resource consumption. Compared to the MHSA and Swin Transformer mechanisms, HDSSM establishes a distinct advantage in small-object localization, elevating AP*_S_* to 0.280. Furthermore, empirical efficiency metrics show that the state-space design manages the computational footprint at a competitive 9.12 GFLOPs. Consequently, LRD-Inst with HDSSM yields a peak inference throughput of 45.4 FPS, demonstrating its viability for deployments with constrained computational budgets while avoiding the latency penalties typically associated with traditional dense attention mechanisms.

**Table 10:** Comparative evaluation of various contextual modeling mechanisms under a constrained parameter budget.

Mechanism	AP	AP_50_	AP_75_	AP_S_	AR	FPS	FLOPs (G)	Params (M)
Standard MHSA	0.564	0.813	0.611	0.274	0.650	39.3	9.31	3.16
Swin Transformer	0.564	0.811	0.610	0.269	0.651	37.5	9.35	3.61
VMamba Block	0.560	0.807	0.608	0.266	0.645	44.1	9.17	3.34
HDSSM (Ours)	0.568	0.815	0.618	0.280	0.653	45.4	9.12	3.44

All configurations are evaluated using identical training hyper-parameters on the self-constructed mixed-apple dataset.

## 5 Discussion

Experimental results show that mitigating orchard visual degradations requires no brute-force parameter scaling. LRD-Inst demonstrates that a target-oriented dual-branch topology—isolating spatial details from global semantic context via FDSP and HDSSM—resolves the intrinsic trade-off between contour delineation and holistic recognition, keeping feature propagation lightweight yet highly expressive. Additionally, the AS-Dice loss proves that scale imbalance can be resolved by reshaping the optimization landscape and gradient distribution rather than stacking heavy multi-scale pyramids. Consequently, LRD-Inst shifts the focus from naive network compression to precision-guided structural allocation, providing an efficient architectural framework tailored for edge-deployed fruit harvesting pipelines while maintaining domain-specific geometric fidelity.

Despite these advancements, certain boundary conditions restrict immediate scaling to broader agricultural scenarios. First, the AS-Dice loss relies on empirical thresholds and static weights, limiting adaptability across varying crop species or fluctuating imaging distances. Second, although complex cluster occlusions and bagging variations captured in the empirical evaluation represent core, ubiquitous challenges in robotic harvesting, the validation remains geographically and taxonomically focused on a single apple variety within a specific harvest window, lacking extensive longitudinal evaluations across multi-species trees or diverse trellis configurations. Finally, the perception pipeline operates within the 2D image plane, yielding planar projection masks lacking explicit volumetric and depth awareness essential for complex physical manipulation.

These constraints define clear trajectories for future research. Cross-domain robustness will be enhanced by expanding the evaluation datasets across diverse pomological categories and geographical regions, concurrently utilizing self-supervised domain adaptation and meta-learning to accommodate broader species variations and multi-seasonal illumination dynamics. Crucially, closed-loop manipulation requires migrating from 2D analysis to 3D spatial understanding. Future work will focus on fusing the structurally optimized features of LRD-Inst with multi-modal LiDAR or RGB-D point clouds for 3D instance segmentation, enabling the vision loop to directly infer precise 3D orientations and volumetric centers, thereby bridging the gap between planar perception and collision-free manipulation in dynamic orchard ecosystems.

## 6 Conclusion

This study presents LRD-Inst, a frequency-decoupled dual-branch instance segmentation framework engineered to tackle severe visual degradations—such as specular reflections from bagged fruits and dense canopy occlusions—inherent in modern unstructured orchards. By integrating State Space Models (SSMs) for long-range semantic recurrence with parallel high-frequency detours for morphological preservation, the architecture resolves the precision-context bottleneck restricting conventional lightweight CNNs. Comprehensive evaluations confirm that LRD-Inst achieves an optimal equilibrium among boundary precision, architectural compactness, and real-time inference speed, outperforming state-of-the-art lightweight paradigms without requiring deep serial bottlenecks. Furthermore, scale-stratified gradient optimization demonstrates that the localization of diminutive targets can be substantially enhanced by reshaping the optimization landscape rather than expanding network capacity. Ultimately, while the empirical validation is currently confined to a single crop variety within a localized seasonal window, the underlying architectural paradigm provides a theoretically grounded and deployable vision framework for agricultural robotics. Future work will expand evaluation fields across diverse agronomic conditions to systematically extend the generalization limits of the framework.

## Data Availability

The raw data supporting the conclusions of this article will be made available by the authors, without undue reservation.
